# Changes in Shoulder Arthroscopy Trends in the Different Health Areas of the Aragon Region of Spain

**DOI:** 10.7759/cureus.92034

**Published:** 2025-09-11

**Authors:** David Araguas-Castillon, Felicito Garcia-Alvarez

**Affiliations:** 1 Surgery, Universidad de Zaragoza, Zaragoza, ESP; 2 Orthopaedics and Traumatology, Hospital Clinico Universitario Lozano Blesa, Zaragoza, ESP

**Keywords:** arthroscopy, epidemiology, health policy, implementation, shoulder

## Abstract

Purpose: The objective of the study was to analyze the epidemiology and implementation of shoulder arthroscopy, with a focus on disparities and differences between public and private hospitals in the different health areas of the Aragon region of Spain.

Methods: A retrospective analysis of the database of the Aragon Institute of Health Sciences from January 1, 1996, to December 31, 2021, was conducted.

Results:During the study period, 17,938 shoulder arthroscopies were performed, increasing the overall number of interventions by 360.6 times. The male/female ratio was 1.25. Mean age increased from 44.3 (± 7.6) years in 1996 to 52.3 (± 13.2) years in 2015 (p<0.001). In the Teruel area, the incidence rate of arthroscopies was 161.5/100,000 inhabitants in 2009; however, it decreased to 45.2 in 2021. On the other hand, in the Zaragoza I area, the number increased from 64.1 in 2009 to 121.5 in 2021. Between 1996 and 2015, 60.3% (2,883 patients) of the total number of private-funded interventions were performed on male patients, whereas 51.14% (3,288 patients) of the total number of public-funded interventions were performed on male patients (p<0.001); since 2016, 58.46% (2,014 patients) of the total number of private-funded interventions were performed on male patients, whereas 53.46% (1,328 patients) of the total number of public-funded interventions were performed on male patients (p<0.001). Mean length of stay was 1.49 (±2.47) days in private-funded admissions versus 1.78 (±1.20) days in public-funded admissions between 1996 and 2015 (p<0.001); since 2016, the were no differences between the two groups. Readmission rate from 1996 to 2015 was higher in private-funded surgeries (553 readmitted patients of 3550, 0.16%) compared to public-funded surgeries (614 readmitted patients of 6237, 0.1%) (p<0.001); since 2016, readmission rate was higher in private-funded surgeries (498 readmitted patients of 3245, 0.15%) compared to public-funded surgeries (150 readmitted patients of 2426, 0.06%) (p<0.001).

Conclusions:Arthroscopic shoulder procedures did not follow the same development and implantation in all the areas of Aragón; there were existing differences depending on the health area. The different idiosyncrasies of public and private hospitals favor the differences between them in terms of implementation and development of shoulder arthroscopy.

## Introduction

In the last 30 years, arthroscopic surgery has experienced great development and has increased in popularity worldwide. This is due to improvements in equipment, instrumentation, and implants, a better surgeon's knowledge, training in arthroscopic techniques, and surgeon and patient interest in more precise and less invasive surgeries [1]. Among its advantages are less invasive approaches, better intra-articular visualization, lower risk of postoperative complications, less pain in the immediate postoperative period, faster recovery, and reduced hospital costs, since many procedures are performed on an outpatient basis. As a result, both surgeons and patients have demonstrated a preference for arthroscopic surgery over open surgery [2]. The development of minimally invasive surgery has involved a decrease in surgical morbidity and mortality. Orthopedic surgery has been part of this trend through arthroscopic techniques for joint surgery. All these reasons contributed to the increase in arthroscopic procedures. The number of knee arthroscopy procedures followed an upward trend over the years [3,4]. The number of arthroscopic meniscectomy procedures in Spain presented an increase of 33% between 2003 and 2018 [4].

Arthroscopic techniques require specific training by the orthopedic surgeon. Due to its high technical requirements, there may be differences in the implementation of shoulder arthroscopy between the different health areas. In fact, time trends and geographical variations in arthroscopy rates have been described among several states of the United States [5]. Hospitals in the Aragon region of Spain vary in the training backgrounds of staff, and specialists frequently move between hospitals. This could contribute to a heterogeneous composition of shoulder arthroscopy training among hospital staff. The different structures of public and private funded hospitals may favor the differences between them in terms of implementation and development of shoulder arthroscopy and in relation to hospital lengths of stay and readmissions. The aim of this study was to analyze the epidemiology and implementation of shoulder arthroscopy, with a focus on disparities and differences between public and private hospitals in different health areas of the Aragon region in Spain.

## Materials and methods

This was a retrospective cross-sectional observational study of anonymized data from the Aragon Institute of Health Sciences (IACS) Big Data project (BIGAN) from the The Minimum Basic Hospital Discharge Dataset, which includes individual anonymized clinical (diagnoses and procedures), demographic (age, gender, and healthcare areas of residence), and administrative data (admission, discharge, main procedure dates, and ambulatory surgery) of all discharges in the regional Health Services hospitals. Data on patients who underwent arthroscopic shoulder surgery in the database from January 1, 1996, to December 31, 2021, were requested. Ethical approval (PI22-520) was obtained for this study from the Research Ethics Committee of Aragon (Comité de Ética de la Investigación de la Comunidad Autónoma de Aragón (CEICA)). Patient consent was waived due to anonymization.

A retrospective review of procedures performed under codes "OR_J4_" and "OR_K4_" defined as right and left shoulder arthroscopy from 2016 to 2021 (using the International Classification of Diseases tenth edition (ICD-10)) [6]; and from 1996 to 2015 under coding "80.21" defined as shoulder arthroscopy (using the International Classification of Diseases ninth edition (ICD-9)) [7] was performed. The data were received in an anonymized manner, by assigning a code to each patient so that they could not be identified and were stored on a password-protected hard disk.

The 2021 population of the Aragon region was 1,331,938 inhabitants [8]. The region is divided into eight health areas (Zaragoza I, Zaragoza II, Zaragoza III, Huesca, Teruel, Barbastro, Calatayud, and Alcañiz). The population corresponding to each health area in 2021 was 198,320 inhabitants for Zaragoza I area, 392,177 inhabitants for Zaragoza II area, 312,217 inhabitants for Zaragoza III area, 114,845 inhabitants for Huesca area, 77,353 inhabitants for Teruel area, 110,772 inhabitants for Barbastro area, 48,785 inhabitants for Calatayud area, and 71,792 inhabitants for Alcañiz area [9]. The study included all subjects, residents and non-residents in Aragon, who underwent shoulder arthroscopy surgery in all the hospitals of Aragon, including both public and private funded hospitals. Patients from healthcare insurances General Mutual Fund of Civil Servants of the State (MUFACE), General Legal Mutual Fund (MUGEJU), and the Social Institute of the Armed Forces (ISFAS) were considered as privately funded.

The demographic variables requested were age, gender, and health area at the time of the intervention. The clinical variables requested were the timeline of intervention, type of funding, procedure performed, main diagnosis, length of stay, and number of readmissions in the subsequent 30 days for all causes associated with the shoulder arthroscopic surgery.

Statistical analysis was performed using Statview-Statgraphics software 5.0.1 (Statgraphics Technologies, Inc., The Plains, Virginia, United States). A basic descriptive analysis was performed. The normality of the distribution was tested. The Chi-square test and Fisher's exact test were used to compare qualitative variables. Analysis of variance (ANOVA), using Snedecor's F distribution, was used for comparison of quantitative data. All data are presented as means, and deviations are presented as standard deviations (SD). A p-value of 0.05 or less was considered a threshold for statistical significance.

## Results

**Figure 1:**
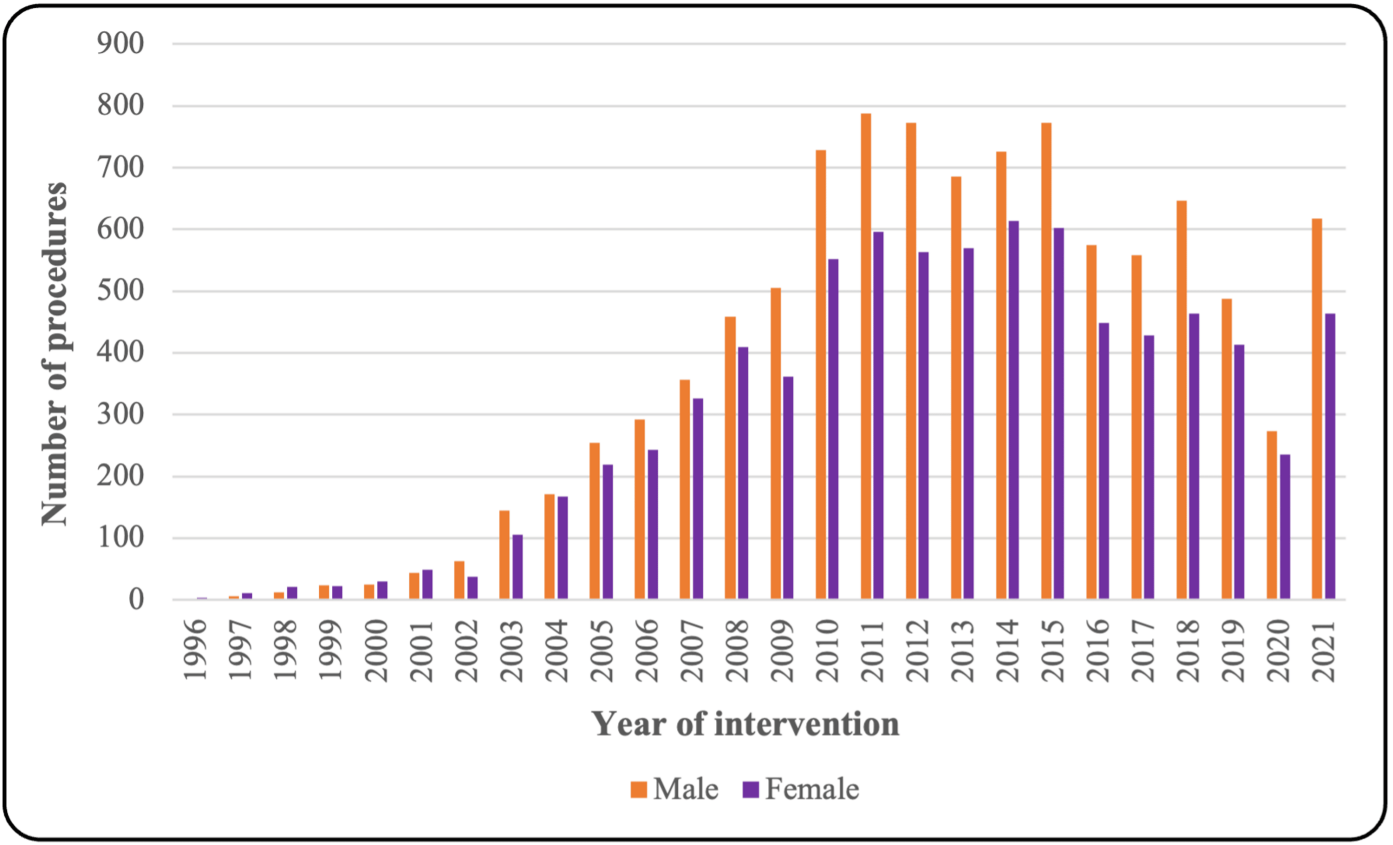
Sex distribution in the study period (1996-2021)

**Figure 2:**
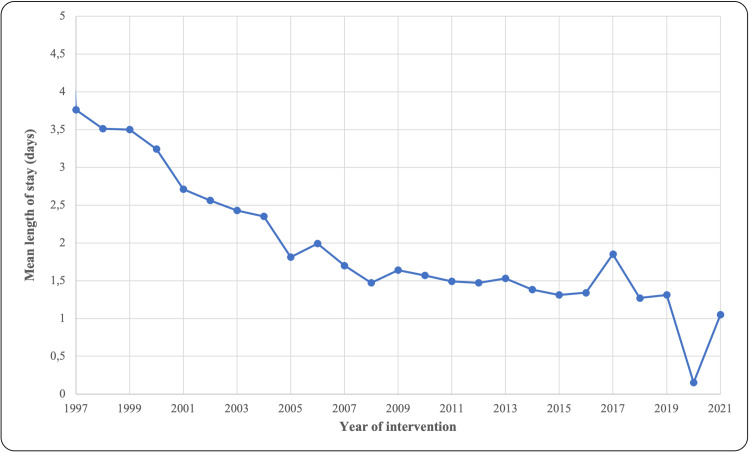
Mean length of stay (days) in 1997-2021

The total number of procedures recorded between January 1, 1996, and December 31, 2021, in Aragon for both public and private funding was 17,938 arthroscopic shoulder surgeries. The overall number of procedures performed between 1996 and 2021 increased by 360.66 times. A total of 9,985 of the 17,938 procedures were performed on male patients, the male/female ratio being 1.25. Figure 1 shows the total annual procedures performed grouped by gender. Significant differences are summarized in Table 1. The mean age of the patients at the time of the intervention was 44.3 (± 7.6) years in 1996, rising to 52.3 (± 13.2) years in 2015 (p<0.001), whereas for the most recent period (2016-2021), there were no differences (p=0.288). Total average length of stay decreased from 3.76 (±1.52) days in 1997 to 1.05 (±0.74) days in 2021, as shown in Figure 2 (p<0.001).

**Table 1:** Summary of significant differences between public-funded and private-funded patients

Parameters	Private Funded	Public Funded	p<0.0001	Test values
Age (years), mean ± SD	In 1996: 44.3 ± 7.6	In 2015: 52.3 ± 13.2	p-value	ANOVA F-Value 4.306
Age (years), 1996-2015, mean ± SD	50.32 ±20.65	51.9 ±13.89	<0.0001	ANOVA F-Value 26.634
Age (years), 2016-2021, mean ± SD	50.69 ±13.59	52.10 ±15.06	0.00017	ANOVA F-Value 14.16
Length of stay (days), mean ± SD	In 1997: 3.76 ±1.49	In 2021: 1.05 ±0.75	9.51 × 10⁻⁷⁶	ANOVA F-Value 396.71
Length of stay (days), 1996-2015, mean ± SD	1.49 ± 2.47	1.78 ± 1.20	<0.0001	ANOVA F-Value 84.145
Length of stay (days), 2016-2021, mean ± SD	1.21 ± 8,72	1.36 ± 4.97	0.658	ANOVA
Readmission rate, first 30 postoperative days, 1996-2015	0.16% (553 of 3550 patients)	0.1% (614 of 6237 patients)	3.85 × 10⁻⁹	Chi² = 38.75
Readmission rate, the first 30 postoperative days, 2016-2021	0.15% (498 of 3245 patients)	0.06% (150 of 2426 patients)	1.04 × 10⁻³⁴	Chi² =156.50
Male%, 1996-2015	60.33% (2883 of 4779 patients)	51.14% (3288 of 6430 patients)	4.68 × 10⁻²²	Chi² = 93.22
Male%, 2016-2021	58.46% (2014 of 3445 patients)	53.46% (1328 of 2484 patients)	0.00014	Chi² = 14.46

**Figure 3:**
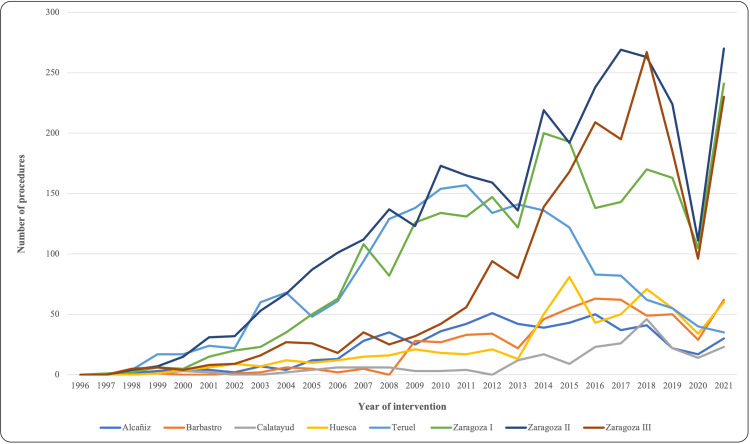
Differences in the implementation of shoulder arthroscopy in the different health areas

**Figure 4:**
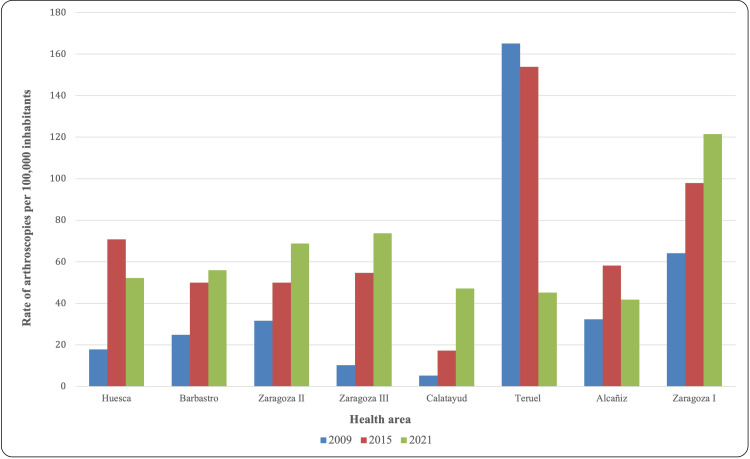
Rate of shoulder arthroscopies per 100,000 inhabitants in the years 2009, 2015, and 2021

Depending on the different health areas, there were existing differences in the implementation of shoulder arthroscopy, as shown in Figure 3. A greater number of shoulder arthroscopies were performed between 2001 and 2013 in the Zaragoza II, Teruel, and Zaragoza I areas. However, in the most recent years (2015-2021), Zaragoza II, Zaragoza III, and Zaragoza I areas accumulated the highest number of shoulder arthroscopic procedures, with a significant increase in the Zaragoza III area from 2013 onwards. An abrupt decrease in activity occurred in 2020 due to the SARS-CoV-2 pandemic, with a subsequent recovery in 2021, with the number of procedures returning to pre-pandemic levels. To contextualize these findings, the number of procedures is shown in Figure 4 in relation to the population belonging to each health area, expressed as the rate of shoulder arthroscopies per 100,000 inhabitants.

**Figure 5:**
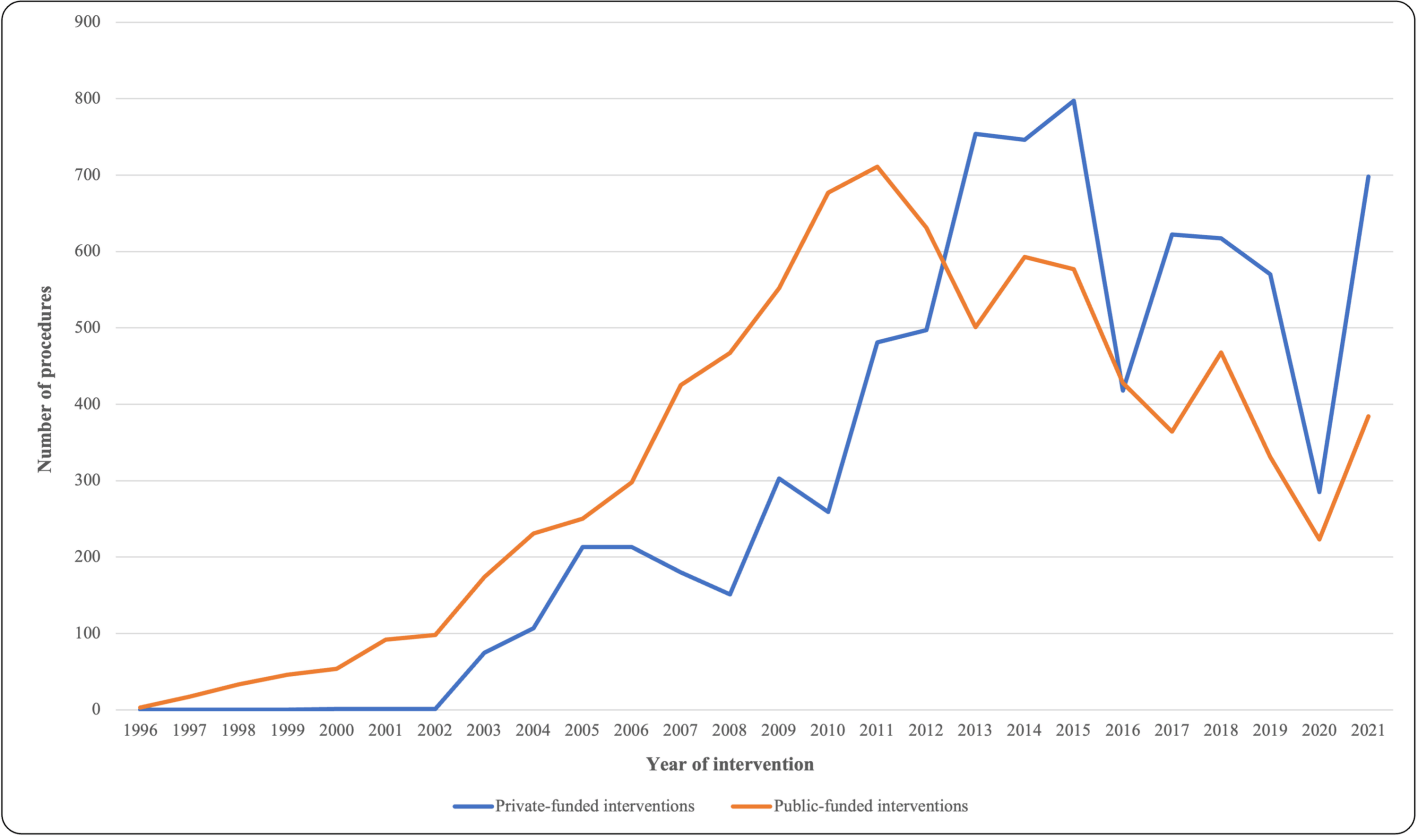
Trends in number of procedures in public and private-funded institutions during the study period (1996-2021)

Regarding the type of funding of the interventions, procedures were more numerous in public-funded hospitals from 1996 to 2012, with a reversal of the trend after that year, with private-funded hospitals accounting for a greater number of interventions from 2013 to 2021, as shown in Figure 5. In total, 8,721 public-funded and 6,995 private-funded interventions were registered. Regarding the distribution by gender according to the type of funding, between 1996 and 2015, 60.3% (2,883 patients) of the total number of private-funded interventions were performed on male patients, whereas 51.14% (3,288 patients) of the total number of public-funded interventions were performed on male patients (p<0.001). Since 2016, 58.46% (2,014 patients) of the total number of private-funded interventions were performed on male patients, whereas 53.46% (1,328 patients) of the total number of public-funded interventions were performed on male patients (p<0.001). The mean age of patients who underwent surgery between 1996 and 2015 was 50.32 (±20.65) years for private-funded patients, while standing at 51.9 (±13.89) years for public-funded patients (p<0.001). The same tendency was observed since 2016, with the mean age being 50.69 (±13.59) years for private-funded patients and 52.10 (±15.06) years for public-funded patients (p<0.001). Mean length of stay was 1.49 (±2.47) days for private-funded admissions versus 1.78 (±1.20) days for public-funded admissions between 1996 and 2015 (p<0.001). Since 2016, there were no differences in length of stay according to the type of funding, which was 1.21 (±8,72) days for private-funded admissions and 1.36 (±4.97) for public-funded admissions (p=0.658). Of the total number of patients who underwent surgery from 1996 to 2021, a small number required readmission during the first 30 postoperative days. Readmission rate from 1996 to 2015 was higher in private-funded surgeries (553 readmitted patients of a total of 3550 private-funded, 0.16%) compared to public-funded surgeries (614 readmitted patients of a total of 6237 public-funded, 0.1%) (p<0.001). Readmission rate since 2016 was higher in private-funded surgeries (498 readmitted patients of 3245, 0.15%) compared to public-funded surgeries (150 readmitted patients of 2426, 0.06%) (p<0.001). 

Since 2016, according to the ICD-10, the most frequent main diagnosis in patients who underwent shoulder arthroscopy were rotator cuff tears (2568 patients, 41.87%), shoulder instability (1174 patients, 19.14%), subacromial impingement syndrome (756 patients, 12.33%), adhesive capsulitis (703 patients, 11.46%), shoulder pain (380 patients, 6.21%), superior labral anterior posterior (SLAP) lesions (274 patients, 4.47%) and other shoulder injuries (278 patients, 4.45%).

## Discussion

Shoulder arthroscopy is a recently implemented complex technique that requires specific training by the orthopedic surgeon. To our knowledge, we did not find a study on the evolution of the implementation of the shoulder arthroscopy technique. These findings from our study may inform policy decisions regarding training allocation, regional resource distribution, and surgical capacity planning. Some authors have described increases in the number of arthroscopic meniscectomies [3,4]. In our study, the total number of procedures followed an upward trend over the years, confirming the trends observed in other joints [3], reaching its peak in 2011. Using a national database of the United States, Ahmed et al. found that arthroscopic Bankart procedures increased from 2009 to 2018, and the number of open Bankart procedures decreased [10].

In our study, the different chronology in the implementation of shoulder arthroscopy according to health areas, as well as the differences between public and private hospitals, are notable. Jain et al. found large geographical variations among several states in the United States for knee arthroscopy (from 63.31 to 721.72 per 100,000 persons), shoulder arthroscopy (from 53.02 to 438.25 per 100,000 persons), and arthroscopic rotator cuff repair (from 11.94 to 185.35) [5].

Saunders et al. surveyed the American Shoulder and Elbow Surgeons society members, and these surgeons related regional disparity in perceived barriers to access for Medicaid health insurance program between the South and the Northeast regions in regard to implant reimbursement at surgeons' primary surgical facility, and this was perceived as a barrier to access in 30% more of the Southern respondents compared to respondents from the Northeast [11]. Nevertheless, in our country, the public health system is free for all the citizens and every regional área has a reference public hospital.

We found that in 2020, there was a downward spike in the total number of procedures performed due to the COVID-19 pandemic; however, in 2021, the number of procedures recovered to 2019 levels. Morrisey et al. reported that United States residents performed significantly fewer shoulder, wrist, knee, and leg/ankle arthroscopies in academic year 2019-20 than in academic year 2018-19 [12].

There was an increase in the mean age of the patients at the time of intervention and a decrease in the mean length of stay due to improvements in equipment, technique, surgeons' training, and a better knowledge of postoperative evolution. The increase in the mean age may be related to the popularization of arthroscopy and a more demanding elderly population in terms of physical well-being. A general decrease in the length of stay has been observed in arthroscopic compared to open interventions in other joints [13,14].

Depending on the health areas, a different timeline in the implementation of shoulder arthroscopy was observed. Regarding the evolution of the rate of arthroscopies per 100,000 inhabitants, the Teruel area suffered a sharp decrease, going from 165.1/100,000 in 2009 to 45.2/100,000 in 2021. On the other hand, the Zaragoza I area experienced the largest increase from 64.1/100,000 in 2009 to 121.5/100,000 in 2021. These changes could be due not only to the natural increase in the implementation of the technique but also to the movement of orthopedic surgeons and management changes in the different health areas. Demographic changes, only by themselves, do not justify such an abrupt difference in such a short period of time.

The results observed in terms of gender and mean age according to the type of funding could be conditioned by the number of interventions carried out through private sports insurances from federations due to sporting accidents, and the greater number of young male athletes. Differences in the mean length of stay between public and private funded hospitals could be due to different protocols employed. Siciliani et al. found that the specialised public treatment centres and private treatment centres have, on average, 18% and 40% shorter length of stay, respectively, compared with NHS public hospitals, and interpreted such differences as because of efficiency as opposed to selection of less complex patients [15]. We have not found any explanation for differences in readmission; it may be in relation to the shorter length of stay of private centers. 

In our study, the proportion of procedures performed on male patients was higher; this could be because factors such as physical activity, sport practiced, or professional occupation played an important role, and the male sex has been associated with increased risk of shoulder-related injuries associated with sports and trauma [16,17]. Data from the United States National Electronic Injury Surveillance System database concluded that younger individuals and males showed greater sport-related injuries, while older populations and females more commonly showed home-related injuries [18]. Nevertheless, some authors found that only 29.3% of the patients who were diagnosed with a primary rotator cuff injury in the New York Statewide Planning and Research Cooperative System (SPARCS) from 2009 through 2018 underwent surgical intervention, and female patients were associated with decreased odds of undergoing rotator cuff surgery [19].

This study had a number of limitations. First, only the Aragon region was studied; demographic differences may produce different results, making it possible that the same results were not obtained in other regions or countries. Second, the ICD-9 classification system, used until 2015, provides less data in terms of classification of diagnoses and procedures performed compared to ICD-10. In future years, homogeneous use of ICD-10 could improve the reliability of the results. Although these limitations do not jeopardize the validity of the study, it would be interesting in terms of perspective to verify these trends in other locations and to extend them to a wider geography, confirming that shoulder arthroscopy is a surgeon-dependent technique. It would be beneficial to promote the training of orthopedic surgeons in shoulder arthroscopy in places where a deficit is detected, as well as to homogenize surgical indications. 

## Conclusions

Shoulder arthroscopy did not follow the same evolution and timeline of implementation in all the health areas of Aragon, experiencing significant decreases or increases in the same area. From 1996 to 2021, the number of interventions increased by 360.6 times in the country, and the mean age increased from 44.3 years in 1996 to 52.3 years in 2015. Male patients accounted for 59.5% of private-funded and 51.8% of public-funded operations. Mean length of stay decreased from 3.76 days in 1997 to 1.05 days in 2021, and was higher for public-funded admissions compared to private-funded admissions.

## References

[1] Sanchez-Sotelo J (2018). Mayo Clinic Principles of Shoulder Surgery. https://academic.oup.com/book/24791.

[2] Jithesh K, Meleppuram JJ, Raju A (2024). All-arthroscopic versus mini-open double row rotator cuff repair - a prospective randomised control study based on functional and radiological outcomes. J Orthop.

[3] Kim S, Bosque J, Meehan JP, Jamali A, Marder R (2011). Increase in outpatient knee arthroscopy in the United States: a comparison of National Surveys of Ambulatory Surgery, 1996 and 2006. J Bone Joint Surg Am.

[4] López-Vega M, Doménech-Fernández J, Peiró S, Ridao-López M (2023). Has arthroscopic meniscectomy use changed in response to the evidence? A large-database study from Spain. Clin Orthop Relat Res.

[5] Jain NB, Peterson E, Ayers GD, Song A, Kuhn JE (2019). US geographical variation in rates of shoulder and knee arthroscopy and association with orthopedist density. JAMA Netw Open.

[6] (2004). World Health Organization: ICD- 10: International statistical classification of diseases and related health problems:tenth revision. ICD- 10: International Statistical Classification of Diseases and Related Health Problems: Tenth Revision, 2nd ed.

[7] (2025). Centers for Disease Control and Prevention: International Classification of Diseases, Ninth Revision (ICD-9). index.

[8] (2023). Government of Aragon: Population and housing census [Website in Spanish]. https://aplicacionesportalaragon.aragon.es/tablas/iaest/areas-tematicas/02-demografia-y-poblacion/01-cifraspoblacion-y-censos/03_censos/03_censo_2021/01-poblacion-pcaxis-2021.html.

[9] (2023). Datasets - Aragón Open Data. (2023). Accessed March 30. Datasets - Aragón Open Data [Document in Spanish].

[10] Ahmed AS, Gabig AM, Dawes A, Gottschalk MB, Lamplot JD, Wagner ER (2023). Trends and projections in surgical stabilization of glenohumeral instability in the United States from 2009 to 2030: rise of the Latarjet procedure and fall of open Bankart repair. J Shoulder Elbow Surg.

[11] Saunders P, Mathur A, Frausto J, Ramirez CD, Khan AZ, Bushnell B, Kassam HF (2025). Regional trends in perceptions of American Shoulder and Elbow Surgeons towards barriers to access for patients with Medicaid: limited perioperative service access, low patient engagement, and decreased reimbursement are regionally consistent obstacles. JSES Rev Rep Tech.

[12] Morrisey Z, Carroll T, Castle P, Botros M, Wilbur D (2024). Trends in orthopaedic surgery resident case volume and the impact of COVID-19 on resident education. J Orthop.

[13] Upfill-Brown A, Shi B, Carter M (2022). Reduced costs, complications, and length of stay after arthroscopic versus open irrigation and débridement for knee septic arthritis. J Am Acad Orthop Surg.

[14] Honnenahalli Chandrappa M, Hajibandeh S, Hajibandeh S (2017). Ankle arthrodesis-open versus arthroscopic: a systematic review and meta-analysis. J Clin Orthop Trauma.

[15] Siciliani L, Sivey P, Street A (2013). Differences in length of stay for hip replacement between public hospitals, specialised treatment centres and private providers: selection or efficiency?. Health Econ.

[16] Ferreira RM, Fernandes LG, Minghelli B, Feito Y, Sampaio AR, Pimenta N (2025). Sport-related injuries in Portuguese CrossFit(®) practitioners and their characteristics. Muscles.

[17] Boufadel P, Fares MY, Daher M, Lopez R, Khan AZ, Abboud JA (2025). Epidemiology of acromioclavicular joint separations presenting to emergency departments in the United States between 2004 and 2023. Shoulder Elbow.

[18] Reiad TA, Peveri E, Dinh PV, Owens BD (2025). Epidemiology of shoulder injuries presenting to US emergency departments. Orthopedics.

[19] Quinn M, Marcaccio SE, Brodeur PG, Testa EJ, Gil JA, Cruz AI Jr (2025). In patients with rotator cuff tears, female, Hispanic, African American, Asian, socially deprived, federally insured, and uninsured patients are less commonly treated surgically. Arthroscopy.

